# Urinary NGAL and KIM-1 in Canine Monocytic Ehrlichiosis

**DOI:** 10.3390/vetsci12020105

**Published:** 2025-02-01

**Authors:** Mariana Elisa Pereira, Darlan Henrique Canei, Yolanda Paim Arruda Trevisan, Fernanda Harumi Maruyama, Nathália de Assis Pereira, Eduarda Pavan, Carolina Zorzo, Adriane Jorge Mendonça, Luciano Nakazato, Domingos Tabajara de Oliveira Martins, Juliano Bortolini, Daniel Moura de Aguiar, Arleana Bom Parto Ferreira de Almeida, Valéria Régia Franco Sousa

**Affiliations:** 1 Postgraduate Program in Veterinary Science, Faculty of Veterinary Medicine, Federal University of Mato Grosso, Avenue Fernando Correa da Costa, Boa Esperança, Cuiabá 78060-900, MT, Brazil; marianaep@gmail.com (M.E.P.); darlancanei@hotmail.com (D.H.C.); yolandapaim@hotmail.com (Y.P.A.T.); fer_19_nanda@hotmail.com (F.H.M.); nathaliaassis89@gmail.com (N.d.A.P.); carolzorzo6@gmail.com (C.Z.); 2 Pharmacology Area, Basic Health Sciences Department, Faculty of Medicine, Federal University of Mato Grosso, Avenue Fernando Correa da Costa, Boa Esperança, Cuiabá 78060-900, MT, Brazil; eduarda.pavan@hotmail.com (E.P.); tabafcm@gmail.com (D.T.d.O.M.); 3 Faculty of Veterinary Medicine, Federal University of Mato Grosso, Avenue Fernando Correa da Costa, Boa Esperança, Cuiabá 78060-900, MT, Brazil; adrianejorge.m@gmail.com (A.J.M.); lucnaka@gmail.com (L.N.); daniel.aguiar@ufmt.br (D.M.d.A.); arleferreira@gmail.com (A.B.P.F.d.A.); 4 Exact and Earth Sciences Institute, Federal University of Mato Grosso, Avenue Fernando Correa da Costa, Boa Esperança, Cuiabá 78060-900, MT, Brazil; julianobortolini@gmail.com

**Keywords:** *Ehrlichia canis*, glomerulonephritis, urinary biomarkers

## Abstract

Glomerulonephritis, caused by the deposition of immune complexes, can lead to kidney damage in dogs with canine monocytic ehrlichiosis (CME). The early diagnosis of renal insult is important to prevent severe kidney disease in infected dogs by *Ehrlichia canis*. This study aimed to investigate urinary biomarkers of renal function, neutrophil gelatinase (uNGAL), and kidney injury molecule-1 (uKIM-1) using the Luminex^®^ xMAP^®^ platform, and the proportion of mixed or high molecular weight proteinuria in dogs with CME. This study included blood samples of thirty dogs with clinical signs of CME and amplified DNA for *E. canis* (CME group) and six dogs of different breeds and both sexes, aged 3 to 7 years, that showed no clinical-laboratory alterations or tick parasitism and were tested negative for *E. canis* via PCR (control group). The total calcium, phosphorus (*p* < 0.05), urea (*p* < 0.001), creatinine (*p* < 0.05), urinary density (*p* < 0.05), urinary protein creatinine ratio (*p* < 0.001), uNGAL (*p* < 0.05), and uKIM-1 (*p* > 0.05), as well as the proportion of high molecular weight proteinuria and mixed proteinuria (*p* < 0.01), were measured. Elevated serum concentrations of creatinine, urea, and phosphorus combined with reduced urinary density, increased urinary creatinine–protein ratio, urinary NGAL, and mixed proteinuria detected renal damage in dogs with CME, while KIM -1 remained unchanged. uNGAL can detect early renal lesions, reflecting renal damage before a significant increase in serum creatinine occurs, and appears to be an early diagnostic biomarker in renal disease in dogs with CME.

## Simple Summary

Canine monocytic ehrlichiosis (CME) is a vector-borne disease associated in many cases with renal dysfunction. The early detection of renal damage using urinary biomarkers is important to slow the progression of chronic kidney disease and decrease the high rates of morbidity and mortality. This study investigated urinary biomarkers of renal function in dogs with monocytic ehrlichiosis, focusing on urinary neutrophil gelatinase-associated lipocalin (uNGAL) and urinary kidney injury molecule-1 (uKIM-1). In 30 dogs with CME and 6 healthy control dogs, total calcium, phosphorus, urea, creatinine, urinary specific gravity, urinary protein creatinine ratio, uNGAL, and uKIM-1 were measured. The findings showed that elevated serum concentrations of creatinine, urea, and phosphorus, along with reduced urinary density and an increased urinary protein–creatinine ratio and uNGAL levels, indicated renal damage in dogs with CME. However, uKIM-1 levels remained unchanged. uNGAL can detect early kidney injuries before significant increases in serum creatinine, making it a valuable early diagnostic biomarker for renal disease in dogs with CME.

## 1. Introduction

Glomerulonephritis due to the deposition of immune complexes can cause kidney damage in dogs infected with canine monocytic ehrlichiosis (CME) [1,2]. The early diagnosis of kidney damage is essential for establishing a therapeutic protocol to prevent the chronic progression of kidney disease and reduce the high rates of morbidity and mortality [3]. The serum concentrations of the biomarkers, urea and creatinine, commonly used as measures of kidney function, change when there is a loss of more than 75% of the renal mass [4], reinforcing the need for more specific parameters for the early diagnosis of kidney injury, in addition to determining the severity and prognosis of the disease [5].

Several studies have investigated the performance of early biomarkers of kidney function, including Neutrophil Gelatinase-Associated Lipocalin (NGAL) and Kidney Injury Molecule 1 (KIM-1) [6,7,8,9,10]. Urinary NGAL (uNGAL) and KIM-1 (uKIM-1) are markers of renal function located in the tubules that are regulated after kidney injury. KIM-1 is used as a marker for acute kidney injury (AKI) in rats, humans, and dogs [5]. NGAL is a protein filtered by the glomeruli and reabsorbed by the renal tubules and is normally expressed at low concentrations, and it is one of the most prominent renal biomarkers [11]. In dogs, uNGAL precedes increases in serum creatinine of acute and chronic kidney injury [4].

Furthermore, the proteomic analysis by electrophoresis of urinary proteins has been used to identify the site of injury in nephrons according to the molecular weight of the proteins [12,13]. Macromolecular proteinuria, the loss of high molecular weight proteins (>60 kDa), is associated with glomerular disease; micromolecular proteinuria, the loss of low molecular weight proteins (<60 kDa), is related to tubular and/or interstitial injury. The loss of both molecular weight proteins reflects glomerular and tubulointerstitial lesions [14].

Therefore, the objectives of this study were to investigate renal function in dogs with CME by analyzing the urinary biomarkers NGAL and KIM-1; the changes in variables routinely used to assess renal function (urea, creatinine, urinary density [USG], and urinary protein–creatinine ratio [UPC]); the proportion of high molecular weight urinary proteins (HMWP; >60 kDa) and mixed proteinuria (MP) (HMWP and low molecular weight urinary proteins; <60 kDa (MP)); and the antibody titers against *E. canis*.

## 2. Materials and Methods

### 2.1. Animals and Clinical Analysis

This study was conducted between June 2018 and February 2020 at the Veterinary Hospital of the Federal University of Mato Grosso, Cuiabá, Brazil. Thirty dogs with clinical signs of monocytic ehrlichiosis and DNA amplification from blood samples for *E. canis* in the PCR were included in this study. The dogs with CME were aged between 1 and 11 years (median 4.5 years), including 18 males and 12 females; 15 were defined breeds, 15 were mixed breeds, and all had a history of tick infestation. Dogs with pyuria were excluded from this study because lower urinary tract infections can increase urinary NGAL concentration. Another exclusion criterion was coinfection with *Leishmania* sp. by PCR [15].

The control group consisted of 6 dogs aged between 3 and 7 years (median 5 years), including 5 males and 1 female, 3 of defined breed and 3 mixed breeds, which had no history of tick parasitism, no clinical or laboratory alterations, and negative PCR for *E. canis*.

A clinical form was filled out for each animal containing information regarding attitude (alertness or apathy), mucosal color (normocolored or hypocolored/icteric), rectal temperature (hypothermia < 37.5 °C; normothermia 37.5 to 39.2 °C; pyrexia > 39.3 °C), and degree of dehydration (5 to 7% mild dehydration; >7% moderate to severe).

In all dogs, blood samples were collected from the external jugular vein. CBC analysis (Poche 100 Hematological Analyzer Sysmex Corporation^®^, Norderstedt, Germany) and serum biochemistry (Wiener^®^ Automated Biochemical Analyzer, Rosario, Argentina) were measured. Five milliliters of urine were collected using cystocentesis after local antisepsis with chlorhexidine. The physicochemical analysis of urine was performed using a Combur-test^®^ reagent strip (Cobas, Roche Diagnostics^®^, Mannheim, Germany). USG was determined using a refractometer (reference range 1.020–1.045). The UPC was measured using the Wiener^®^ Automated Biochemical Analyzer (UPC reference value < 0.2) after centrifuging the total urine at 712× *g*.

### 2.2. DNA Extraction and PCR for E. canis

DNA was extracted from 250 µL of the blood samples using the phenol–chloroform–isopropanol method [16] and subsequently quantified in a NanoDrop™ 2000/2000c Spectrophotometers (Thermo Fisher Scientific™, Thermo Fisher Scientific Inc., Waltham, MA, USA). DNA was detected using primers E_can0503F (5′-CAG CAA ATT CCA ATC TGC ACT TC-3′) and E_can0503R (5′-GAG CTT CCA ATT GAT GGGTCT G-3′), in which the Ecaj_0503 gene encoded 147 pairs of bases of a hypothetical protein [system E_can0701]. PCR analysis was performed, with adaptations, in an Applied Biosystems VeritiTM 96-Well thermocycler (Model #:9902, Thermo Fisher Scientific Inc., Waltham, MA, USA) with 4 µL of dNTP, 2 µL of 10× PCR buffer [Sigma, St. Louis, MO, USA], 0.5 µL of 10 pmol primer, 0.2 µL of Taq DNA polymerase [Sigma-Aldrich], and 17.3 µL of Milli-Q water for each DNA sample, which ensured at least 100 ng of DNA template per reaction. The reaction program followed that described by Socolovschi et al. [17]. A positive control sample from an *E. canis* dog was placed and a negative control was included. The amplified products were separated by electrophoresis in a 2% agarose gel for 1 h at 60 V, stained using Gel Red (Biotium Inc., Fremont, CA, USA), and visualized using a ChemiDocTM XRS + (Hercules, CA, USA) device and ImageLabTM^®^ software version 6.0.1.

### 2.3. Indirect Immunofluorescence Assay (IFA)

IFA was performed using the Cuiabá 16 strain of *E. canis* as an antigen [18]. The serum was diluted from 1:40 in a ratio of two to the final titer. Negative control and positive control were included on each slide. A rabbit anti-dog IgG conjugate (whole molecule-fluorescein isothiocyanate, F7884-2mL, Sigma Diagnostics, St. Louis, MO, USA) was added at a dilution of 1:900. The slide was then covered with glycerol and examined under an epifluorescence microscope (40× objective).

### 2.4. Urinary Protein Electrophoresis

After urine collection, the samples were centrifuged and the supernatant was separated for urinary protein determination by Bradford method (Coomassie brilliant blue G-250, Sigma^®^) and for electrophoresis analysis by sodium dodecyl sulfate polyacrilamide gel electrophoresis (SDS-PAGE) [19]. Quantification was performed using a spectrophotometry system at a wavelength of 595 nm (Epoch™ Multi-volume Spectrophotometer System BioTek^®^–Gen5™ Data Analysis Software). The minimum and maximum absorbance of the protein concentration was generated and the samples that did not remain within this range were diluted in milli Q water or precipitated with trichloroacetic acid. Thus, the amount of protein present in the urine applied to the gel was standardized at 5 μg/well. For assembly and electrophoretic running of the gels, the Mini-protean II TETRA CELL^®^ system was used according to the manufacturer’s instructions. To identify the proteins, markers with molecular weights of 22,000, 100,000, 60,000, 45,000, 30,000, 20,000, 12,000, and 8000 Da were used. SDS-PAGE gels were run in a pH 8.4 buffer solution at an initial current of 30 V and increased to 40 V at the end of the run. Gel images were captured and processed using a photo documentation system (ChemiDoc XRS+, Bio-Rad, Hercules, CA, USA). Visual assessment and comparison with the pre-stained electrophoretic molecular weight marker ColorBurstTM (Sigma-Aldrich) and the migration distance in the gel were used to estimate the molecular weight of each protein band. For a better interpretation, two categories were considered: MP (mixed proteinuria) and HMWP (high molecular weight urinary proteins).

### 2.5. Measurement of Urinary NGAL and KIM-1

The renal biomarkers were measured from urine supernatant (approximately 200 µL) previously stored at −80 °C. Urinary NGAL and uKIM-1 were determined (average of triplicates) using the MILLIPLEX^®^ MAP KIT Canine Kidney Toxicity Expanded Magnetic Bead Panel 1 immunoassay methodology on the Luminex^®^ xMAP^®^ platform (EMD Millipore Corporation, Billerica, MA, USA). The intra- and inter-assay variability was less than 10% and 15%, respectively.

### 2.6. Statistical Analysis

The normality of variables was verified by Shapiro–Wilk test. The variables with normal distribution were analyzed by paired t-test and those that did not present a normal distribution were analyzed by Mann–Whitney test using the Past statistical program [20].

As no normality was observed in the distribution of urea, creatinine, USG, UPC, uNGAL, and uKIM-1 residues, the data were analyzed using Generalized Additive Models of Location, Scale, and Shape (GAMLLS). Additionally, data were subdivided into three groups according to the UPC (<0.2 (non-proteinuric), between 0.2 and 0.5 (borderline proteinurics), and >0.5 (proteinurics)) and the behavior of the uNGAL and uKIM-1 biomarkers was compared between the groups. The Akaike information criterion (AIC) was used to evaluate the different fitted distributions. The model with the lowest value for this criterion was chosen. The degree of correlation between the variables was based on Spearman’s correlation. *p* < 0.05 was considered statistically significant. HMWP with MP in dogs with naturally acquired CME was compared using the chi-square test of equality of proportions.

## 3. Results

The dogs with CME were aged between 1 and 11 years (median 4.5 years), 60% were males (18), 50% were of a defined breed (15), and all had a history of tick infestation. Regarding the clinical signs of dogs with CME, eight dogs presented with apathy (27%), twenty-three had hypocolored and/or jaundiced mucous membranes (77%), two presented with pyrexia, nine presented with hypothermia (7% and 30%, respectively), and eight presented with moderate to severe dehydration (27%).

One dog infected with CME did not show seroconversion, while the others had antibody titers ranging from 40 to 81,920. In dilutions 1:40 and 1:320, there was one dog in each; 1:640, 1:1280, 1:40,960, and 1:81,920 had two dogs each; 1:2560 had five dogs; and 1:5120 and 1:20,480 had four dogs. Finally, at a dilution of 1:10,240, there were six dogs.

Dogs infected with CME had significantly lower erythrocyte counts than the control group (*p* < 0.05), and twenty-five dogs with CME had anemia (Table 1). Twenty-three dogs presented with thrombocytopenia, reaching 2. 10^3^ cel/µL; one had platelet aggregates and six had thrombocytosis. There were no statistically significant differences in the serum total calcium and creatinine concentrations between groups (*p* > 0.05) (Table 1). Serum phosphorus concentrations were significantly higher in infected dogs than in the control (*p* < 0.05). Proteinuria was present in 70% of CME dogs and borderline in 16.6% (*p* < 0.05). The proportion of HMWP was 6.67% and the proportion of MP was 93.3% in dogs infected with CME (*p* < 0.01).

Urinary NGAL concentrations were significantly higher in dogs with *E. canis* infection (*p* < 0.05). No significant differences in the concentration of uKIM-1 were observed between the infected and control dogs (*p* > 0.05). The mean uNGAL and uKIM-1 were significantly higher in dogs infected with CME and borderline proteinuria. Dogs with CME and proteinuria had higher mean uNGAL and lower mean uKIM-1 levels (Table 2). There was a moderate positive correlation between urea and creatinine (r = 0.620, *p* < 0.001), uNGAL (r = 0.398, *p* = 0.036), and UPC (r = 0.621, *p* < 0.001); creatinine and UPC (r = 0.397, *p* = 0.029); USG and uKIM-1 (r = 0.416, *p* = 0.022); and uNGAL and UPC (r = 0.467, *p* < 0.001). There was a moderate negative correlation between urea and USG (r = −0.448, *p* = 0.016); urea and uKIM-1 (r = −0.525, *p* < 0.001); USG and UPC (r = −0.507, *p* < 0.001); uNGAL and uKIM-1 (r = −0.365, *p* = 0.047); and uKIM-1 and UPC (r = −0.362, *p* = 0.049).

## 4. Discussion

In the CME subclinical phase, there are high and persistent titers of antibodies that can induce glomerulonephritis by the deposition of immune complexes [2]. It can also be reinforced by higher mean values of uNGAL in dogs infected with CME and the high titers of antibodies against *E. canis* found in this study. The dogs that progress to the chronic phase of the disease have high antibody titers and are pancytopenic with several clinical signs [21]. Only one dog infected with CME showed no anti-*E. canis* antibodies, probably due to a recent infection without sufficient time for seroconversion [22].

There was a predominance of normocytic normochromic anemia in infected dogs; this finding is reported in both CME [23] and chronic kidney disease [24]. Although the mean platelet value of dogs with CME did not differ significantly from the control group, it was lower than the reference value. Thrombocytopenia can be observed in all phases of the disease [25]. Mild hyperphosphatemia in dogs may be an indication of initial kidney injury as hyperphosphatemia can occur in acute and chronic kidney disease (CKD). As CKD progresses, there are hyperphosphatemia and hypocalcemia; the decline in the glomerular filtration rate induces phosphorus retention, which reduces the concentration of ionized calcium and impairs the formation of calcitriol, leading to hypocalcemia [26].

Although the mechanism of renal damage is not yet fully understood in CME, the predominance of MP in the dogs in this study suggests that in addition to glomerulonephritis, tubulointerstitial lesions occur [27,28]. Furthermore, immunological and inflammatory mechanisms provide infiltration by leukocytes into the renal parenchyma during the acute phase of the disease, resulting in vasculitis [29]. Additionally, an elevation of urinary biomarkers, such as cystatin B and clusterin, in the acute phase of CME suggests subclinical renal injury [30].

The increase in uNGAL levels occurs because a greater portion of NGAL produced by the body is lost by injured glomeruli, like other plasma proteins [31]. The correlation between uNGAL levels and the degree of proteinuria in humans [31] corroborates the higher uNGAL averages in borderline and proteinuric dogs found in our study. Therefore, it is possible to infer that the higher mean levels of uNGAL in borderline and proteinuric dogs help with their prognosis since high concentrations of uNGAL are associated with death in dogs with chronic kidney disease [32]. uKIM-1 is a highly specific biomarker for AKI [7], which may explain the higher mean uKIM-1 levels in borderline proteinuric dogs than in proteinuric dogs. The increase in uNGAL levels probably reflects glomerular and tubular kidney damage [31] that could be related to the glomerulonephritis and vasculitis described in dogs infected with *E. canis* [33].

The moderate correlation between uNGAL and UPC associated with increased uNGAL and the marked mixed proteinuria observed in dogs with CME support the use of these renal biomarkers in the investigation of renal function in this disease. Although UPC is cheaper than uNGAL measurement, uNGAL may be more sensitive, especially in detecting moderate renal injury [34]. The measurement of symmetric dimethylarginine (SDMA), which is a biomarker of renal function used to diagnose CKD, was not performed, and we consider this as a limitation of this study, notwithstanding that SDMA remained unchanged in dogs with renal injury in acute CME [30].

## 5. Conclusions

Dogs with CME showed increased uNGAL levels even when serum urea and creatinine concentrations remained unchanged, demonstrating the importance of this biomarker in detecting early CKD. Significant increases in phosphorus, urea, and UPC levels, a decrease in USG levels, and a predominance of MP were observed, reinforcing the impairment of renal function in those with CME. There was no significant increase in the concentration of KIM-1.

## Figures and Tables

**Table 1:** Mean and standard deviation, median, and AIC of hematological, serum, and urinary biochemical variables, uNGAL and KIM-1, of control (CG) and CME dogs (CMEG).

Variable	Reference Range	Mean (SD)		Median (Min–Max)		AIC	
		CG	CMEG	CG	CMEG	CG	CMEG
Erythrocyte (10^3^ cel/µL)	5.5–8.5	7.26 ± 0.86	2.75 ** ± 2.24	6.97 (6.26–8.6)	1.69 (0.71–8.17)	-	-
Hematocrit (%)	37–55	49.31 ± 4.24	19.07 ** ± 14.5	48.5 (44.9–56)	12.75 (5.6–55.8)	-	-
Hemoglobin (g/dL)	12–18	17.11 ± 1.16	6.14 ** ± 4.87	17.3 (15.6–18.6)	3.75 (1.7–17.9)	-	-
Leukocyte (10^3^ cel/µL)	6–17	10.56 ± 4.28	17.92 ± 19.94	9.5 (6.7–18.9)	10.8 (1.6–92.6)	-	-
Monocytes (10^3^ cel/µL)	1–4.8	2.06 ± 0.67	0.66 ± 0.51	2.0 (1.3–3.0)	0.6 (0–1.3)	-	-
Platelet (10^3^ cel/µL)	200–500	308 ± 108.27	142.26 ± 150.99	281 (204–470)	102 (2–680)	-	-
Calcium (mg/dL)	9–11.3	6.65 ± 4.02	8.35 ± 0.97	8.25 (1.2–10.6)	8.25 (6.7–10.1)	-	-
Phosphorus (mg/dL)	2.6–6.2	3.65 ± 1.79	6.29 * ± 2.65	3.7 (0.8–6.1)	5.4 (12.5–125.9)		
Urea (mg/dL)	21.4–59.92	39.16 ± 5.84	116.71 ** ± 107.02	41.5 (30–45.0)	75.0 (12.0–415.0)	7.0	111.75
Creatinine (mg/dL)	0.5–1.5	1.13 ± 0.19	1.62 ± 1.74	1.1 (0.9–1.4)	1.0 (0.2–7.9)	0.28	0.60
USG (g/L)	1.015–1.045	1.035 ± 11.69	1.023.9 * ± 13.6	1.032.5 (1.022–1.056)	1.018 (1.010–1.052)	8.75	16.50
UPC (mg/dL)	<0.2	0.07 ± 0.11	3.77 ** ± 4.34	0.04 (0.003-0.30)	2.25 (0.04–17.7)	0.04	4.73
uKIM-1 (pg/mL)	-	165.62 ± 37.16	120.61 ± 131.70	169.11 (117.37–215.4)	90.95 (2.1–475.22)	49.91	130.85
uNGAL (ng/mL)	-	0.24 ± 0.15	7.6 *^†^ ± 3.57	0.22 (0.07–0.53)	8.71 (0.27–13.56)	0.12	2.50

SD: Standard Deviation; AIC: Akaike information criterion; USG: urinary density; uKIM-1: urinary Kidney Injury Molecule 1; uNGAL: urinary Lipocalin Associated with Neutrophil Gelatinase; UPC: urinary creatinine–protein ratio; * *p* < 0.05 compared to reference value; ** *p* < 0.001 compared to reference value; *^†^ *p* < 0.05 compared to control group.

**Table 2:** Mean values of uNGAL and uKIM-1 in dogs with CME according to the UPC subdivision.

UPC	uNGAL	uKIM-1
<0.2	2.31	160.58
0.2–0.5	7.18	257.51
>0.5	8.71	80.41

uNGAL: urinary Lipocalin associated with Neutrophil Gelatinase; uKIM-1: urinary Kidney Injury Molecule 1.

## Data Availability

If requested, the authors will provide the data supporting this study’s interpretations.
